# Identifying SM-miRNA associations based on layer attention graph convolutional network and matrix decomposition

**DOI:** 10.3389/fmolb.2022.1009099

**Published:** 2022-11-23

**Authors:** Jie Ni, Xiaolong Cheng, Tongguang Ni, Jiuzhen Liang

**Affiliations:** School of Computer Science and Artificial Intelligence and Aliyun School of Big Data and School of Software, Changzhou University, Changzhou, China

**Keywords:** microRNA, small molecule, deep learning, association prediction, matrix decomposition

## Abstract

The accurate prediction of potential associations between microRNAs (miRNAs) and small molecule (SM) drugs can enhance our knowledge of how SM cures endogenous miRNA-related diseases. Given that traditional methods for predicting SM-miRNA associations are time-consuming and arduous, a number of computational models have been proposed to anticipate the potential SM–miRNA associations. However, several of these strategies failed to eliminate noise from the known SM-miRNA association information or failed to prioritize the most significant known SM-miRNA associations. Therefore, we proposed a model of Graph Convolutional Network with Layer Attention mechanism for SM-MiRNA Association prediction (GCNLASMMA). Firstly, we obtained the new SM-miRNA associations by matrix decomposition. The new SM-miRNA associations, as well as the integrated SM similarity and miRNA similarity were subsequently incorporated into a heterogeneous network. Finally, a graph convolutional network with an attention mechanism was used to compute the reconstructed SM-miRNA association matrix. Furthermore, four types of cross validations and two types of case studies were performed to assess the performance of GCNLASMMA. In cross validation, global Leave-One-Out Cross Validation (LOOCV), miRNA-fixed LOOCV, SM-fixed LOOCV and 5-fold cross-validation achieved excellent performance. Numerous hypothesized associations in case studies were confirmed by experimental literatures. All of these results confirmed that GCNLASMMA is a trustworthy association inference method.

## 1 Introduction

As a form of non-coding RNA (ncRNA), MicroRNA (miRNA), is roughly 22 nucleotides in length (Bartel, 2004; Hammond, 2015; Lu and Rothenberg, 2018). Lin-4 was the first human miRNA identified in1993 by Lee *et al.* in Caenorhabditis elegans (Lee et al., 1993; Wightman et al., 1993). With the advent of high-throughput sequencing technologies, an increasing number of miRNAs with important functions in human gene expression have been identified (Denzler et al., 2016; Tagliafierro et al., 2017; Thomou et al., 2017; Gam et al., 2018; Ghini et al., 2018; Liu et al., 2018). Specifically, miRNAs can attach to the 3′ UnTranslated Region (3’ UTR) of target messenger RNAs (mRNAs) via base-pairing to control the degradation of target mRNAs and limit the translation of target mRNAs, hence regulating gene expression (Gorbea et al., 2017). In the control of target mRNA gene expression by miRNA, one miRNA may regulate many target mRNAs, or numerous miRNAs regulate one target mRNA (Saikia et al., 2020; Iwata et al., 2021; Zhong et al., 2021). Several studies demonstrated the role of miRNAs in the maturation of immune cells (Kumar Kingsley and Vishnu Bhat, 2017). Since the profound impact of miRNAs on biological development became apparent, numerous miRNA types have been identified to be involved in biological evolutionary processes (Rupaimoole and Slack, 2017; Cristino et al., 2019).

Small Molecule (SM) drugs are mostly composed of molecules with molecular weights typically fewer than 1,000 g/mol. More than 98 percent of today’s drugs are SMs (Geng and Craig, 2021). The development of SMs that target miRNAs is a current trend in drug research (Dai and Tan, 2015; Yu et al., 2020). In previous drug development, protein enzymes and receptors were typically employed as therapeutic targets. Over 80 percent of drug development was intimately tied to protein enzymes and receptors (Deyle et al., 2017; Yekkirala et al., 2017; Nair et al., 2018; Lai-Kwon et al., 2021). In recent years, more scientific experiments have proven inextricable linkages between SMs and miRNAs (Healy et al., 2012; Monroig et al., 2015; Haniff et al., 2021). When miRNAs fail to regulate the gene expression of an organism, specific disorders such as cardiovascular diseases, neurological diseases and cancers may develop (Kumari et al., 2018; Xia et al., 2019; Dragomir et al., 2021). In addition, SMs are effective in regulating miRNA dysregulation to treat linked endogenous disorders, and numerous SMs have been created for clinical therapy of these diseases (Dragomir et al., 2021).

The development of novel SMs is facilitated by the accurate identification of miRNA-related SMs. Recent studies have focused on discovering possible associations between SMs and miRNAs (Chen et al., 2021; Li et al., 2021; Wang et al., 2021). Early identification approaches used high-throughput screening methods, such as mass spectrometry, fluorescence and reporter genes (Seth et al., 2005; Parsons et al., 2009; Carnevali et al., 2010; Chen et al., 2012). The most frequent method for discovering potential SM-miRNA associations is the reporter genes. On the basis of the reporter genes, a functional novel drug screening method capable of screening lead compounds was proposed. By substituting biomacromolecules with tiny organic compounds, the screening process for drugs could be expedited dramatically. The use of tiny organic compounds throughout the screening procedure could provide information on the functional responses of cells. (Wen et al., 2015). In drug screening research, luciferase reporter genes satisfy the requirements for high sensitivity, target specificity and high throughput (Thorne et al., 2010).

However, it was discovered that biological screening approaches are stochastic and time-consuming. With the proliferation of bioinformatics databases, the number of known SM-miRNA associations increased, as did the calculational methodologies for SM and miRNA similarity. Consequently, machine learning techniques obtained more precise prediction outcomes (Qu et al., 2019). Bioinformaticians have begun to employ machine learning techniques to predict probable SM-miRNA associations to circumvent time-consuming and labor-intensive biological investigations (Wang and Chen, 2019; Wang et al., 2019).

Among the previous methods for predicting probable SM-miRNA associations, (Qu et al., 2018), developed a model titled Triple Layer Heterogeneous Network based Small Molecule-MiRNA Association prediction (TLHNSMMA). TLHNSMMA first merged the known SM-miRNA associations, SM similarity and miRNA similarity into a three-layer heterogeneous network. The three-layer heterogeneous graph was then implemented with an iterative updating algorithm. Finally, the reconstructed SM-miRNA association matrix was obtained using an iterative propagation approach that made extensive use of global data. Based on the establishment of a three-layer SM-miRNA heterogeneous network, (Liu et al., 2020), suggested a novel model for potential SM-miRNA association prediction called Random Walk with Negative Samples (RWNS). Firstly, RWNS obtained integrated similarities of SM and miRNA. Then, Liu *et al.* devised a Credible Negative Sample extraction method (CNSMiRS) to extract plausible negative SM-miRNA samples under the premise that dissimilar SMs/miRNAs are unlikely to be associated with each other’s related miRNAs/SMs. Finally, the reconstructed SM-miRNA association matrix was obtained by implementing a random walk algorithm on the constructed small molecule-disease-miRNA association network. However, the performance of TLHNSMMA and RWNS is dependent on the known SM-miRNA association adjacency matrix. Consequently, (Yin et al., 2019), suggested a model of Sparse Learning and Heterogeneous Graph Inference for Small Molecule-MiRNA Association prediction (SLHGISMMA). Yin *et al.* first used matrix decomposition on known SM-miRNA associations to obtain the new SM-miRNA associations. Then, the new SM-miRNA associations, integrated miRNA similarity and integrated SM similarity were incorporated into a heterogeneous network. Finally, the reconstructed SM-miRNA association matrix was obtained using heterogeneous graph inference. Chen et al. (2021) recently proposed the Bounded Nuclear Norm Regularization for SM–miRNA Associations prediction (BNNRSMMA), which treated the problem of potential SM-miRNA association prediction as a matrix complementation problem. In addition, BNNRSMMA included a regularization term to remove the negative effects of data noise.

In recent years, improvements have been made to machine learning techniques, and deep learning has emerged as one of the brightest new stars (Wang et al., 2020). Deep learning has achieved exceptional results in traditional classification tasks, such as handwritten font recognition (Singh et al., 2021), computer vision (Borges Oliveira et al., 2021) and computational biology (Angermueller et al., 2016). In addition, deep learning has substantially affected the field of potential association prediction. For example, zeng *et al.* proposed a computational framework termed AOPEDF based on drug-target network and deep forest algorithm to predict potential drug-target associations (Zeng et al., 2020). AOPEDF attained excellent performance in identifying molecular targets among known drugs on two external validation datasets by comparison to other machine learning methods. Therefore, we proposed a model of Graph Convolutional Network with Layer Attention mechanism for SM-MiRNA Association prediction (GCNLASMMA). To evaluate the performance of GCNLASMMA, we used two types of cross validation, namely, 5-fold cross-validation and Leave-One-Out Cross Validation (LOOCV). Additionally, we also utilized two types of case studies to confirm the effectiveness of GCNLASMMA in identifying potential miRNAs for investigated SMs. The results showed that GCNLASMMA could accurately and effectively predict the SM-miRNA pairs most likely to be potentially associated.

## 2 Materials and methods

### 2.1 SM-miRNA associations

We named two datasets used in our work after dataset1 and dataset2. Eight hundred and thirty-one SMs in dataset1 were downloaded from three databases, namely SM2miR, DrugBank (Knox et al., 2011) and PubChem (Wang et al., 2009). Five hundred and forty-one miRNAs were downloaded from four databases, namely SM2miR, HMDD (Li et al., 2014), miR2Disease (Jiang et al., 2009) and PhenomiR (Ruepp et al., 2010). Six hundred and sixty-four known SM-miRNA associations were downloaded from a database, namely SM2miR V1.0 (Liu et al., 2013). On the basis of dataset1, we removed the SMs and miRNAs that did not constitute any known association. Then, we obtained dataset2 which included 286 different miRNAs, 39 different SMs and 664 known SM-miRNA association pairs. Specifically, the known SM-miRNA association $A_{ij}$ between the $i_{th}$ SM and the $j_{th}$ miRNA was stored as follows.

### 2.2 Integration of SM similarities

The integrated SM similarity was calculated by (Lv et al., 2015). In his method, a total of four SM similarities were used, namely SM side effect similarity (Gottlieb et al., 2011), gene functional consistency-based similarity for SMs (Lv et al., 2012), SM chemical structure similarity (Hattori et al., 2003) and disease phenotype-based similarity for SMs (Gottlieb et al., 2011). In Lv’s article, the side effect properties of SM were first downloaded from SIDe Effect Resource (SIDER) and calculated by Jaccard score to obtain SMs side effect similarities (Gottlieb et al., 2011). The calculation of gene functional consistency-based similarities for SMs was implemented on the target genes of SMs obtained from the DrugBank and Therapeutic Targets Database (TTD) (Liu et al., 2011). The Gene Set Functional Similarity (GSFS) method was given in the previous article (Lv et al., 2012). Specifically, we downloaded the SM chemical structure information. Then, a graph-based method, SIMilar COMPound (SIMCOMP) (Lv et al., 2012), was applied to obtain SMs’ chemical structure similarities. Finally, the disease phenotype-based similarities for SMs were obtained by calculating the data downloaded from the DrugBank and TTD with the Jaccard score method.

After obtaining all four SM similarities, we named them after SS1 , SS2 , SS3 and SS4 , respectively. Then, the scores of the four SM similarities were integrated by the following formula,

$$SSM=\frac{\sum_{i}\alpha_{i}SSi}{\underset{i}{\sum }\alpha_{i}},\left(i=1,2,3,4\right)$$ (1)

where α represents the weights of SM similarities. All of the measures are important in terms of biology. Thus, we set the values of all α to 1, which means that each SM similarity made an equal contribution to constituting the integrated SM similarity (Li et al., 2004). Finally, the integrated SM similarity $SSM\left(s_{i},s_{j}\right)$ between the $i_{th}$ and $j_{th}$ SMs was obtained after normalization as follows.

$$SSM\left(s_{i},s_{j}\right)=\frac{SSM\left(s_{i},s_{j}\right)}{\sqrt{\sum_{l=1}^{ns}SSM\left(s_{i},s_{l}\right)}\sqrt{\sum_{l=1}^{ns}SSM\left(s_{l},s_{j}\right)}}$$ (2)

### 2.3 Integration of miRNA similarities

Two miRNA similarities, gene function consistency-based similarity (Lv et al., 2012) and indication phenotype-based similarity (Gottlieb et al., 2011), were used to obtain integrated miRNA similarity. Specifically, we downloaded the target scores of each miRNA from the database TargetScan (Agarwal et al., 2015) and obtained gene function consistency-based similarity using the GSFS method (Lv et al., 2012). The indication phenotype-based similarity was obtained from the Human MicroRNA Disease Database (HMDD) version 2.0 (v 2.0), miR2Disease and PhenomiR databases using the GSFS method. Then, we combined the gene function consistency-based similarity and the indication phenotype-based similarity using the Jaccard score. Then, we named the two kinds of miRNA similarities after SM1 and SM2 , respectively. Moreover, the integrated miRNA similarity SMR was obtained by the following equation,

$$SMR=\frac{\sum_{j}\beta_{j}SMj}{\underset{j}{\sum }\beta_{j}},\left(j=1,2\right)$$ (3)

where $\beta_{1}$ and $\beta_{2}$ represent the weights of miRNA similarities. Also, we set the values of $\beta_{1}$ and $\beta_{2}$ to 1, which means each miRNA similarity made an equal contribution to constituting the integrated miRNA similarity. Finally, the integrated miRNA similarity $SMR\left(m_{i},m_{j}\right)$ between the $i_{th}$ and $j_{th}$ miRNAs was obtained after normalization as follows.

$$SMR\left(m_{i},m_{j}\right)=\frac{SMR\left(m_{i},m_{j}\right)}{\sqrt{\sum_{l=1}^{nm}SMR\left(m_{i},m_{l}\right)}\sqrt{\sum_{l=1}^{nm}SMR\left(m_{l},m_{j}\right)}}$$ (4)

### 2.4 GCNLASMMA

**FIGURE 1:**
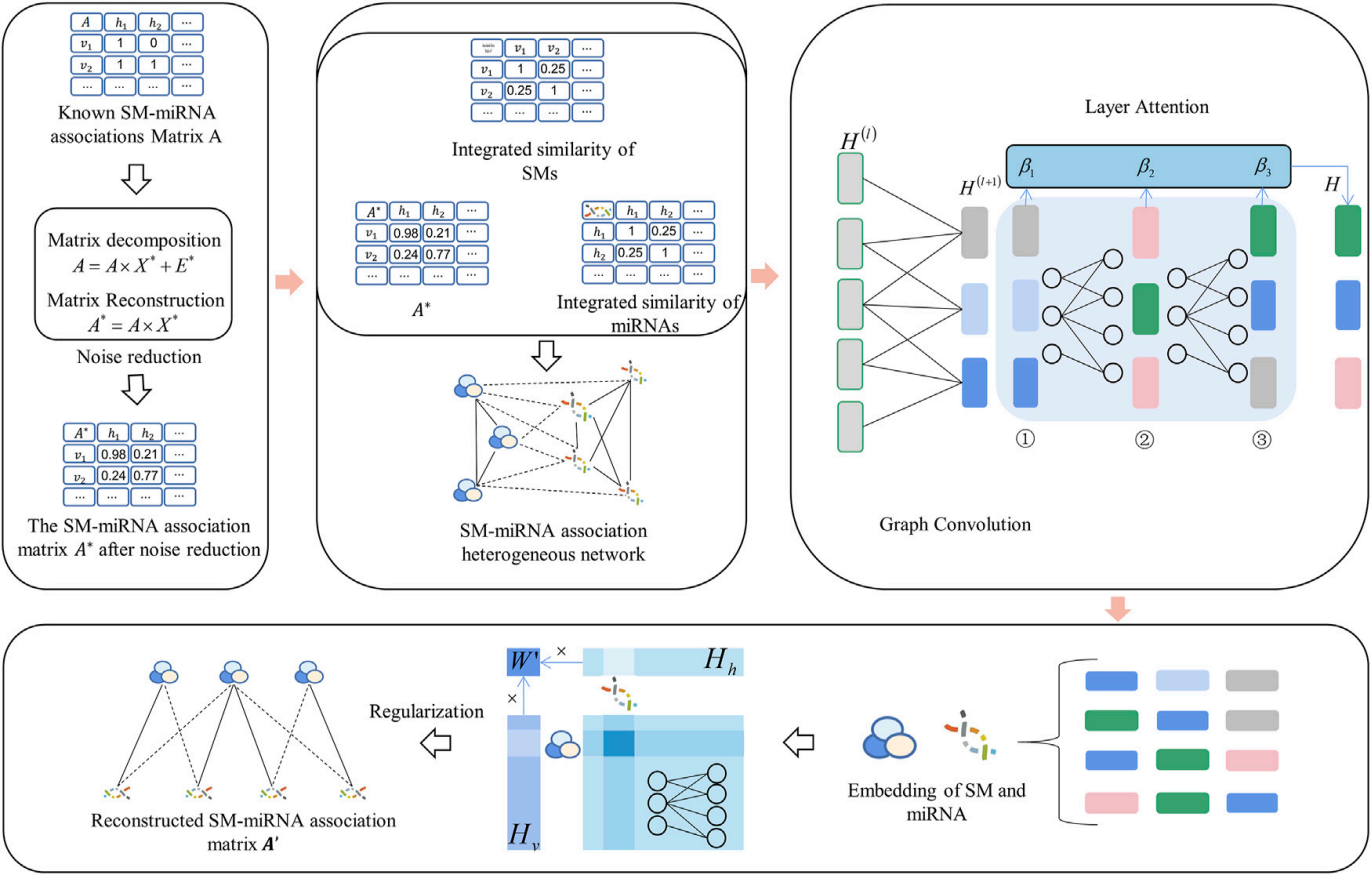
The flow chart of potential SM-miRNA association prediction based on GCNLASMMA. Firstly, the matrix decomposition is applied to obtain the new SM-miRNA associations. Then the new SM-miRNA associations, integrated SM similarity and integrated miRNA similarity are constructed into an SM-miRNA association heterogeneous network. Finally, a graph convolutional network with layer attention mechanism is applied to obtain the reconstructed SM-miRNA association matrix.

GCNLASMMA was separated into two steps. The known SM-miRNA association A was initially decomposed and reconstructed to obtain the new SM-miRNA association $A^{*}$ . The reconstructed SM-miRNA association matrix $A^{\prime }$ was then obtained by calculating the new SM-miRNA association $A^{*}$ using a graph convolutional network with an attention mechanism. More specifically, we obtained the new SM-miRNA associations by matrix decomposition. Then, the new SM-miRNA association matrix, integrated SM similarity and integrated miRNA similarity were constructed into a heterogeneous network. Finally, the graph convolutional network with layer attention mechanism was applied to obtain the reconstructed SM-miRNA association matrix. GCNLASMMA is a model of a neural network with more hidden layers than other networks. The multi-layer calculation thoroughly considered the known features and avoided overfitting. Moreover, the attention mechanism extracted significant information from each layer, thereby improving the accuracy of association prediction (Niu et al., 2021). The specific flow chart of GCNLASMMA is shown in Figure 1.

#### 2.4.1 Matrix decomposition

The existence of noise in known SM-miRNA associations tends to reduce prediction accuracy. Prior research has demonstrated that hidden features with considerable value can be extracted by applying dimension-reduction and noise-reduction to the data (Vidal, 2011). A low-rank matrix is a tool for efficiently obtaining hidden features with significant values (Peng et al., 2012). Therefore, we used matrix decomposition to learn a low-rank matrix from the known SM-miRNA association A . The decomposition of A was performed as follows:

A=A×X+E (5)

Since the above equation contains an infinite number of solutions, we applied the constraint to turn it into:

$$\min_{X,E}{\left\|X\right\|}_{*}+\alpha {\left\|E\right\|}_{2,1}\;s.t.\;A=A\times X+E$$ (6)

where ${\left\|X\right\|}_{*}=\sum_{i}\sigma_{i},\left(\sigma_{i}\;is\;the\;singular\;value\;of\;matrix\;X\right)$ , ${\left\|E\right\|}_{2,1}=\sum_{j=1}^{n}\sqrt{\sum_{i=1}^{m}{\left(E_{ij}\right)}^{2}}$ . In Eq. 6, the nuclear norm and sparse norm were applied to constrain X and E , which allowed X and E to be low-rank and sparse matrices, respectively. The balance parameter of low-rank and sparse matrices α was set to 0.1. According to earlier research, if A in Eq. 6 is transformed into an identity matrix, then the model is degenerated to the Robust Principal Component Analysis (RPCA), a convex optimization problem with constraints (Chandrasekaran et al., 2009).

$$\min_{X,E,J}{\left\|J\right\|}_{*}+\alpha {\left\|E\right\|}_{2,1}\;s.t.\;A=A\times X+E,X=J$$ (7)

Based on the previous work (Meng et al., 2014), Eq. 7 can be converted into an unconstrained optimization problem. Therefore, the problem can be resolved using the Exact Augmented Lagrange Multipliers (EALM) algorithm.

$$L={\left\|J\right\|}_{*}+\alpha {\left\|E\right\|}_{2,1}+tr\left(Y_{1}^{T}\left(A-A\times X-E\right)\right)+tr\left(Y_{2}^{T}\left(X-J\right)\right)$$

$$+\frac{\delta }{2}\left({\left\|A-A\times X-E\right\|}_{F}^{2}+{\left\|X-J\right\|}_{F}^{2}\right)$$ (8)

In Eq. 8, the penalty parameter δ≥0 . According to the Inexact Augmented Lagrange Multipliers (IALM) algorithm (See Table 1), we fixed other variables and solved the minimum value of J , X and E by updating the Lagrange multipliers $Y_{1}$ and $Y_{2}$ . Moreover, we defined $X^{*}$ and $E^{*}$ as the solution of Eq. 8. $X^{*}$ represents the similarity matrix of miRNA or SM. $E^{*}$ represents the noise matrix. Then, the new SM-miRNA association $A^{*}$ was expressed as:

$$A^{*}=A\times X^{*}$$ (9)

**TABLE 1:** The illustration of the IALM algorithm.

Algorithm: Inexact augmented lagrange multipliers
Input: Known SM-miRNA association matrix A and α=0.1
Initialize: $X=0,\;E=0,\;Y_{1}=0,\;Y_{2}=0,\;\mu =10^{-4},\;\max_{\mu }=10^{10},\;\rho =1.1,\;\varepsilon =10^{10}$
While true
1. Fix others and $J=argmin\frac{1}{\mu }{\left\|J\right\|}_{*}+\frac{1}{2}{\left\|J-\left(X+Y_{2}/\mu \right)\right\|}_{F}^{2}$
2. Fix others and $X=\left(I+A^{T}A\right)\left(A^{T}A-A^{T}E+J+\left(A^{T}Y_{1}-Y_{2}\right)/\mu \right)$
3. Fix others and $E=argmin\frac{\alpha }{\mu }{\left\|E\right\|}_{2,1}+\frac{1}{2}{\left\|E-\left(A-AX+Y_{1}/\mu \right)\right\|}_{F}^{2}$
4. Update $Y_{1}=Y_{1}+\mu \left(A-AX-E\right)$ *;* $Y_{2}=Y_{2}+\mu \left(X-J\right)$
5. Update $\mu =\min\left(\rho \mu ,\max_{\mu }\right)$
If ${\left\|A-AX-E\right\|}_{\infty }<\varepsilon$ *and* ${\left\|X-J\right\|}_{\infty }<\varepsilon$
End while
Output: $X^{*}$ and $E^{*}$

#### 2.4.2 SM-miRNA heterogeneous network

In this study, the new SM-miRNA association $A^{*}$ , integrated SM similarity SSM and integrated miRNA similarity SMR were combined into a heterogeneous network. There would be a known association between the $i_{th}$ SM and the $j_{th}$ miRNA if element $A_{ij}^{*}$ in $A^{*}$ equaled 1. SSM(i,j) represented the integrated similarity between the $i_{th}$ SM and the $j_{th}$ SM. SMR(i,j) represented the integrated similarity between the $i_{th}$ miRNA and the $j_{th}$ miRNA. The specific equation of the heterogeneous network $A_{H}$ construction is as follows:

$$A_{H}=\left[\begin{array}{cc} ∼SMR & A^{*}\\ A^{*T} & ∼SSM \end{array}\right]$$ (10)

where $A^{*T}$ represents the transpose matrix of $A^{*}$ . In Eq. 10, we normalized the similarity matrix of SM and miRNA by $∼SSM=D_{s}^{-\frac{1}{2}}SSMD_{s}^{-\frac{1}{2}}$ and $∼SMR=D_{m}^{-\frac{1}{2}}SMRD_{m}^{-\frac{1}{2}}$ , respectively. Specifically, $D_{s}=diag\left(\sum_{j}{SSM}_{ij}\right)$ and $D_{m}=diag\left(\sum_{j}{SMR}_{ij}\right)$ .

#### 2.4.3 Graph convolutional network

As classic network models, Long-Short Term Memory (LSTM) and Convolution Neural Network (CNN) are only applicable to grid-structured data. Nevertheless, the Graph Convolutional Network (GCN) can manage data with generalized topological graph structures and deeply explore the features of the data (Habib and Qureshi, 2020). In this paper, we constructed GCNLASMMA, which is a model for graph convolution of biological information. Specifically, GCN was implemented on the SM-miRNA heterogeneous network $A_{H}$ that was constructed by the known SM-miRNA associations, SM similarities and miRNA similarities. GCN is a neural network structure consisting of an input layer, an output layer and many hidden layers that can represent nodes in a low-dimensional manner. Each hidden layer of GCN takes the output of the previous layer as input. The graph convolutional network propagation rule is as follows:

$$H^{\left(l+1\right)}=f\left(H^{\left(l\right)},G\right)=\sigma \left(D^{-\frac{1}{2}}GD^{-\frac{1}{2}}H^{\left(l\right)}W^{\left(l\right)}\right)$$ (11)

In Eq. 11, $H^{\left(l\right)}$ and $H^{\left(l+1\right)}$ denote the embeddings of nodes in the $l_{th}$ and ${\left(l+1\right)}_{th}$ layers, respectively. $D=diag\left(\sum_{j}G_{ij}\right)$ is a diagonal matrix of input graph G , $W^{\left(l\right)}$ represents the trainable weight matrix with a layer-specific value, σ(∙) denotes the nonlinear activation function.

In the encoder part, to learn low-dimensional representations of miRNAs and SMs, we combined the new SM-miRNA association, integrated SM similarity and integrated miRNA similarity into SM-miRNA association heterogeneous network $A_{H}$ . Firstly, we set a penalty factor μ in the input graph G during the propagation process as follows:

$$G=\left[\begin{array}{cc} \mu ∼SMR & A^{*}\\ A^{*T} & \mu ∼SSM \end{array}\right]$$ (12)

Then, we initialized the input layer embeddings as:

$$H^{\left(0\right)}=\left[\begin{array}{cc} 0 & A^{*}\\ A^{*T} & 0 \end{array}\right]$$ (13)

In this way, we obtained the propagation formula for the first layer from Eqs 11, 13:

$$H^{\left(1\right)}=\sigma \left(D^{-\frac{1}{2}}GD^{-\frac{1}{2}}H^{\left(0\right)}W^{\left(0\right)}\right)$$ (14)

In Eq. 12, $W^{\left(0\right)}$ is a weight matrix that acts only between the input layer and the first hidden layer. $H^{\left(1\right)}$ is the first-layer embeddings of the heterogeneous network $A_{H}$ , k is the dimension of the embeddings. Similarly, the propagation rules for the subsequent layers of the GCN encoder followed Eq. 11, where l=1,2,⋯,L . After L iterations, we obtained L k−dimensional embeddings from different graph convolution layers. Exponential linear elements were used as nonlinear activation functions in the graph convolution layer, which sped up the learning process and significantly improved the generalization performance.

In addition, we tried several different combinations of parameters from the range α∈{400, 600, 800, 1000} , lr∈{0.00700, 0.00725, 0.00750, 0.00775, 0.00800} . By adjusting the parameters empirically, we set the dimensions of embeddings k=64 , the number of layers L=3 , the initial learning rate of optimizer lr=0.00725 , the total training epochs α=600 , the two dropout rates β=0.6 and γ=0.4 , the penalty factor μ=6 on both dataset1 and dataset2.

#### 2.4.4 Layer attention mechanism

In addition, the layer attention mechanism was added to this model by introducing an attention mechanism between each layer and storing the position information in $A_{H}$ . As a help for the attention mechanism, we extracted the pertinent information straight from the source data when constructing the embeddings of each layer output during the decoding process. Through this mechanism, we obtained the final SM embeddings and final miRNA embeddings from the fully connected layer: $\left[\begin{array}{c} H_{m}\\ H_{s} \end{array}\right]=\sum a_{l}H^{l}$ , where $H_{m}$ represents the final embeddings of miRNA, $H_{s}$ is the final embeddings of SM. The neural network automatically adjusted the value of $a_{l}$ by the initial input value $\frac{l}{\left(l+1\right)},l=1,2,\cdots ,L$ . Finally, we obtained the reconstructed SM-miRNA association matrix $A^{\prime }$ by an activation function as follows,

$$A^{\prime }=sigmoid\left(H_{m}W^{\prime }H_{s}^{T}\right)$$ (15)

where $W^{\prime }$ is a trainable matrix. The corresponding element $A_{ij}^{\prime }$ is the potential correlation score between miRNA $m_{i}$ and SM $s_{j}$ .

## 3 Results

To evaluate the performance of GCNLASMMA, we used two types of cross validation, namely, 5-fold cross-validation and Leave-One-Out Cross Validation (LOOCV). The two different datasets include the same known 664 SM-miRNA associations. Specifically, dataset 1 has 831 SMs and 541 miRNAs. On the basis of dataset1, we removed the SMs and miRNAs that did not constitute any known association. Then, we obtained dataset2 which has only 286 different miRNAs, 39 different SMs. In this study, the Area Under the receiver operating characteristic Curves (AUCs) obtained under 5-fold cross-validation based on dataset1 and dataset2 were 0.9721 ± 0.0018 and 0.8393 ± 0.0047, respectively. The global AUC and local AUC obtained under LOOCV based on dataset1 were 0.9751 (global LOOCV), 0.9746 (miRNA-fixed LOOCV) and 0.5014 (SM-fixed LOOCV), respectively. Based on dataset2, the AUCs of GCNLASMMA were 0.8504 (global LOOCV), 0.8490 (miRNA-fixed LOOCV) and 0.6398 (SM-fixed LOOCV), respectively. Additionally, we utilized two types of case studies to confirm the effectiveness of GCNLASMMA in identifying potential miRNAs for investigated SMs. Specifically, GCNLASMMA has predicted the potential miRNAs associated with 5-Fluorouracil (5-Fu, CID: 3385), 5-Aza-2′-deoxycytidine (5-Aza-CdR, CID: 451668) and 17β-Estradiol (E2, CID: 5757). For 5-Fu, the results showed that 9, 16 and 39 out of the top 10, 20 and 50 potential related miRNAs in the first type of case studies, 8, 15 and 39 out of the top 10, 20 and 50 potential related miRNAs in the second type of case studies were validated in other literature or databases, respectively. For 5-Aza-CdR, the results showed that 8, 13 and 26 out of the top 10, 20 and 50 potential related miRNAs in the first type of case studies, 8, 14 and 28 out of the top 10, 20 and 50 potential related miRNAs in the second type of case studies were validated in other literature or databases, respectively. For E2, the results showed that 6, 14 and 29 out of the top 10, 20 and 50 potential related miRNAs in the first type of case studies, 4, 11 and 29 out of the top 10, 20 and 50 potential related miRNAs in the second type of case studies were validated in other literature or databases, respectively.

### 3.1 Performance evaluation

In 5-fold cross-validation, all known SM-miRNA associations were randomly separated into five subsets of nearly comparable size. Then, each subset was in turn considered as the test sample, and the rest four subsets were treated as training samples. Moreover, all unknown SM-miRNA pairs were regarded as candidate samples. Subsequently, we obtained a predicted association score matrix by GCNLASMMA, and ranked the scores of each test sample against those of the candidate samples. This partition-prediction-ranking procedure was repeated 100 times to obtain a sound estimate of the mean and variance of GCNLASMMA’s prediction accuracy. Finally, the prediction of a test sample was deemed successful if the sample’s rank was higher than the given threshold. Therefore, we utilized the threshold to calculated the false positive rate (FPR, 1-specificity) and the true positive rate (TPR, sensitivity). The FPR and TPR represented the percentage of candidate samples that lower than the threshold and the percentage of test samples that higher than the threshold, respectively. Then, we regarded FPR and TPR as horizontal and vertical axis. The Receiver Operating Characteristic (ROC) curve were plotted. Finally, we attained the Area Under the ROC Curve (AUC) by computing the area under the ROC curves. In this investigation, GCNLASMMA achieved the AUCs of 0.9721 ± 0.0018 and 0.8393 ± 0.0047 under 5-fold cross-validation based on dataset1 and dataset2, respectively.

LOOCV was further classified as either global and local. Then, the local-LOOCV was subdivided into miRNA-fixed LOOCV and SM-fixed LOOCV. In LOOCV, each known SM-miRNA association was in turn considered to be the test sample and the others were treated as the training samples. Moreover, all unknown SM-miRNA pairs were treated as candidate samples. In miRNA-fixed LOOCV and SM-fixed LOOCV, test samples and training samples were chosen similarly. However, in SM-fixed LOOCV, only unknown SM-miRNA pairs containing the selected SM were regarded as candidate samples. Similarly, in miRNA-fixed LOOCV, candidate samples only included those involving the chosen miRNA. Then, we ranked the score of the test sample against those of the candidate samples. Finally, the prediction of a test sample was deemed successful if the rank of this test sample was higher than the given threshold. Based on dataset1, GCNLASMMA attained the AUCs of 0.9751, 0.9746 and 0.5014 under global LOOCV, miRNA-fixed LOOCV and SM-fixed LOOCV, respectively. Based on dataset2, GCNLASMMA attained the AUCs of 0.8504, 0.8490 and 0.6398 under global LOOCV, miRNA-fixed LOOCV and SM-fixed LOOCV, respectively.

**FIGURE 2:**
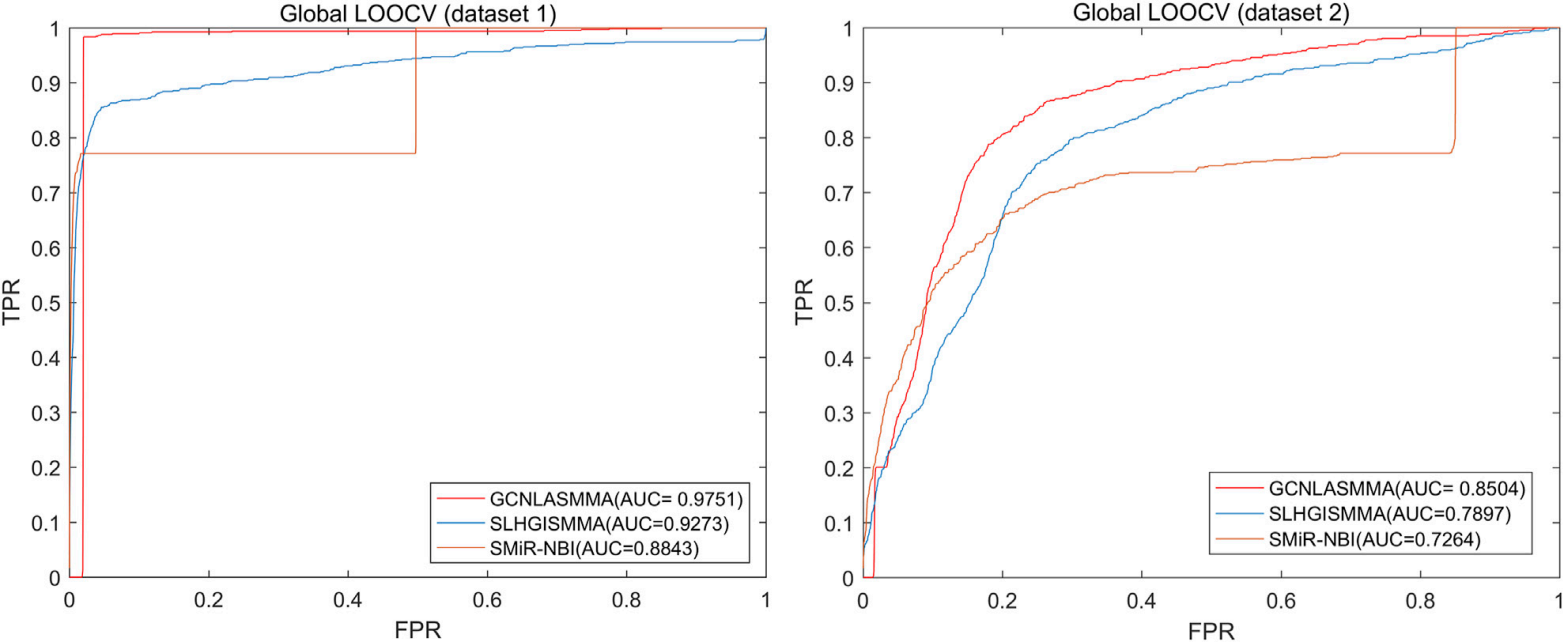
The left half of the figure shows the comparison of GCNLASMMA with two comparison algorithms under global LOOCV based on dataset1. As a result, GCNLASMMA, SLHGISMMA and SMiR-NBI achieve AUCs of 0.9751, 0.9273 and 0.8843, respectively. The right half of the figure shows the comparison of GCNLASMMA with two comparison algorithms under global LOOCV based on dataset2. As a result, GCNLASMMA, SLHGISMMA and SMiR-NBI achieve AUCs of 0.8504, 0.7897 and 0.7264, respectively.

**FIGURE 3:**
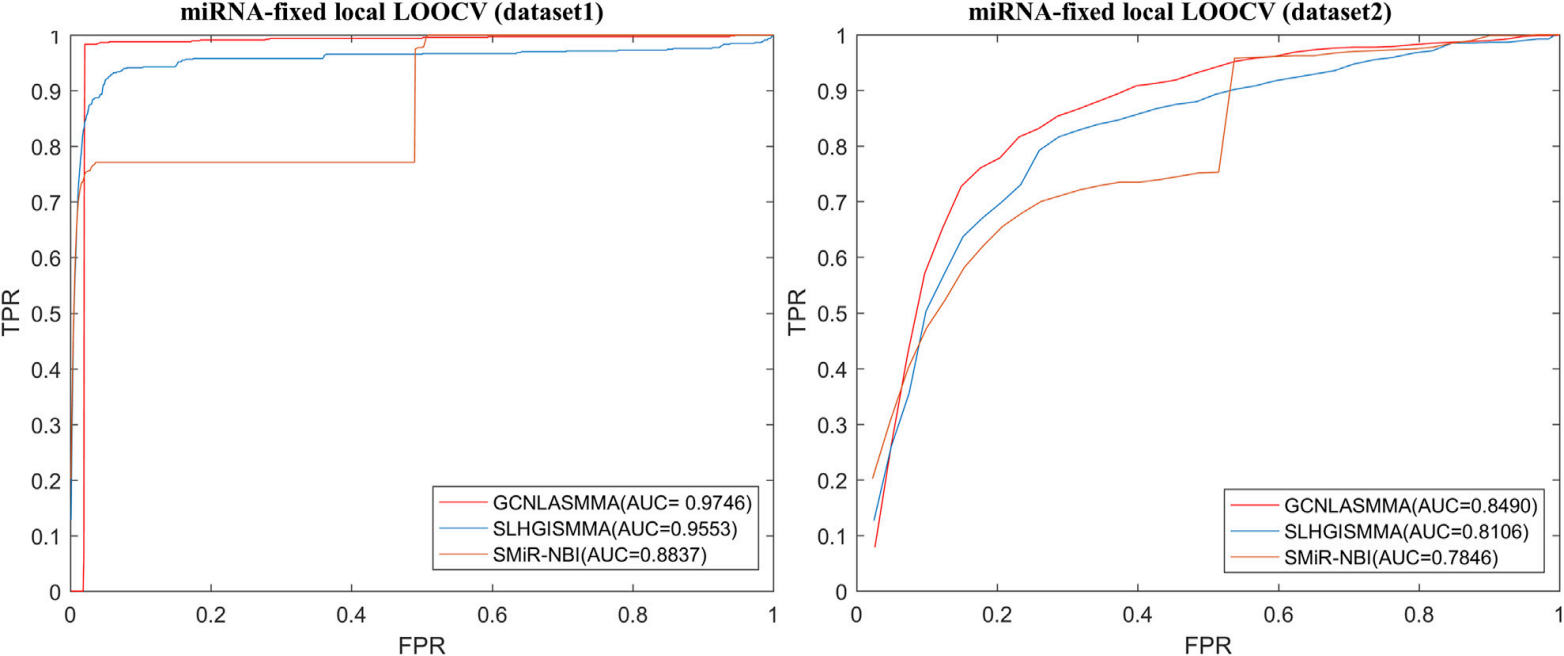
The left half of the figure shows the comparison of GCNLASMMA with two comparison algorithms under miRNA-fixed LOOCV based on dataset1. As a result, GCNLASMMA, SLHGISMMA and SMiR-NBI achieve AUCs of 0.9746, 0.9553 and 0.8837, respectively. The right half of the figure shows the comparison of GCNLASMMA with two comparison algorithms under miRNA-fixed LOOCV based on dataset2. As a result, GCNLASMMA, SLHGISMMA and SMiR-NBI achieve AUCs of 0.8490, 0.8106 and 0.7846, respectively.

**FIGURE 4:**
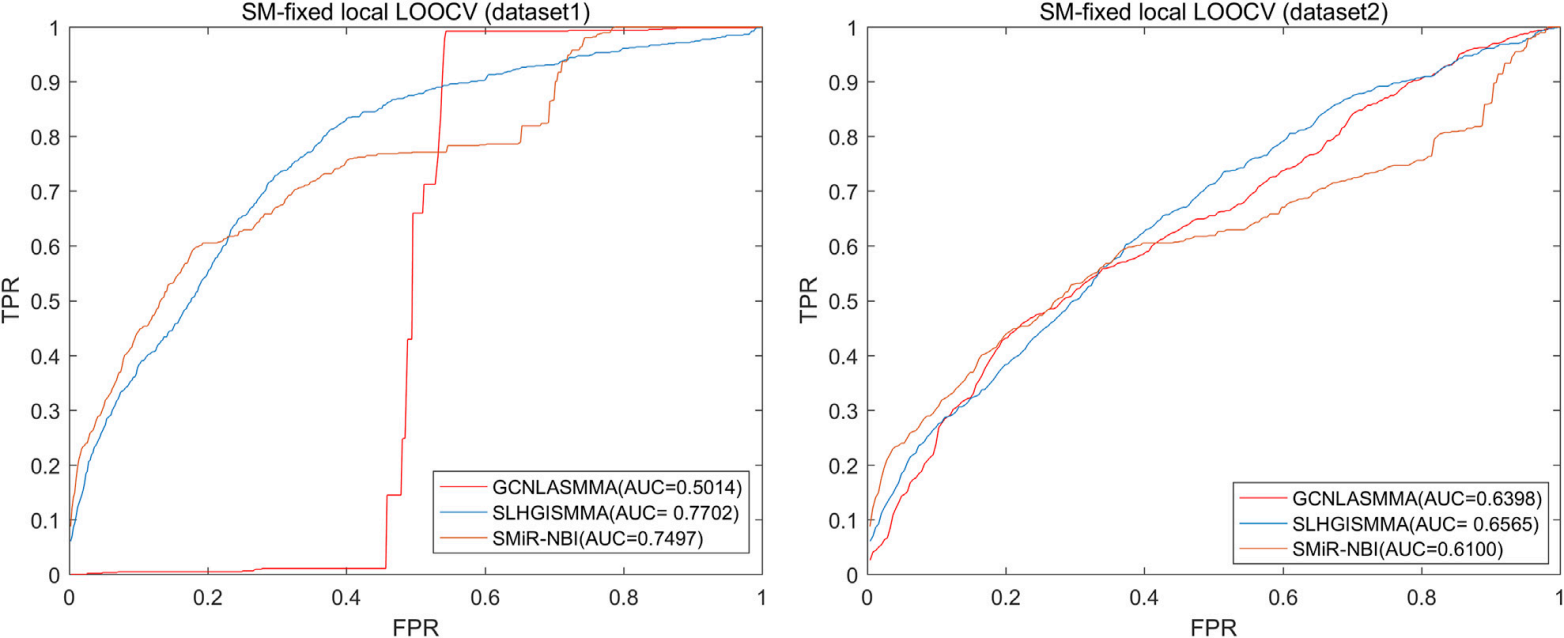
The left half of the figure shows the comparison of GCNLASMMA with two comparison algorithms under SM-fixed LOOCV based on dataset1. As a result, GCNLASMMA, SLHGISMMA and SMiR-NBI achieve AUCs of 0.5014, 0.7702 and 0.7497, respectively. The right half of the figure shows the comparison of GCNLASMMA with two comparison algorithms under SM-fixed LOOCV based on dataset2. As a result, GCNLASMMA, SLHGISMMA and SMiR-NBI achieve AUCs of 0.6398, 0.6565 and 0.6100, respectively.

The AUC comparison figures based on dataset1 (dataset2) were plotted to determine the differences between GCNLASMMA and other models’ outcomes. AUC = 0.5 would suggest that the model was only capable of random prediction, whereas AUC = 1 would indicate that all test samples were accurately predicted. Figure 2 demonstrates that the results of GCNLASMMA under global LOOCV are significantly better than that of SMiR-NBI. Figures 3, 4 show that the results of GCNLASMMA under miRNA-fixed local LOOCV and SM-fixed local LOOCV were significantly better than those of SLHGISMMA and SMiR-NBI. Furthermore, the AUC of miRNA-fixed local LOOCV based on dataset1 is 0.9746, which means almost all potential SM-miRNA associations in dataset1 were predicted successfully.

### 3.2 Case studies

To further illustrate the GCNLASMMA’s applicability to identify potential miRNAs, we conducted two types of case studies on three essential SMs, namely 5-Fluorouracil (5-Fu, CID: 3385), 5-Aza-2′-deoxycytidine (5-Aza-CdR, CID: 451668) and 17β-Estradiol (E2, CID: 5757). On the basis of all known SM-miRNA associations, the first type was applied to forecast potential miRNAs for investigated SMs. As the training set, we utilized the known SM-miRNA associations from dataset1. Then, for each investigated SM, we ranked all candidate miRNAs according on their predicted scores. The second type was used to forecast potential miRNAs for investigated SMs without any known SM-miRNA association. Therefore, we removed all verified associations related to the investigated SMs before the prediction and ranked them as the first type of case studies. After ranking all candidate miRNAs for each investigated SM based on their predicted scores, the top 50 predicted miRNAs were picked out and verified in other literature or databases. Moreover, we selected 10, 20 and 50 associations randomly from all potential associations to further demonstrate the validity of GCNLASMMA. The results show that only 0, 0 and 2 out of random 10, 20 and 50 associations are confirmed in other literature or databases (See Table 2), which significantly worse than the top 10, 20 and 50 miRNAs related to investigated SMs.

**TABLE 2:** Validation of the random 50 SM-miRNAs associations. The first column records the random 1–25 associations. The second column records the random 26–50 associations.

SM	miRNA	Evidence	SM	miRNA	Evidence
CID 4116	hsa-mir-329-2	unconfirmed	CID 2662	hsa-mir-330	unconfirmed
CID 60726	hsa-mir-216b	unconfirmed	CID 7028	hsa-mir-592	unconfirmed
CID 4760	hsa-mir-520c	unconfirmed	CID 5656	hsa-mir-646	32083545
CID 3052	hsa-mir-193a	unconfirmed	CID 3520	hsa-mir-1266	unconfirmed
CID 444036	hsa-mir-199a-2	unconfirmed	CID 43008	hsa-mir-519a-1	unconfirmed
CID 3198	hsa-mir-216a	unconfirmed	CID 3343	hsa-mir-1469	unconfirmed
CID 157922	hsa-mir-1260a	unconfirmed	CID 5566	hsa-mir-548a-3	unconfirmed
CID 3698	hsa-mir-2110	unconfirmed	CID 5493444	hsa-mir-1285-2	unconfirmed
CID 4212	hsa-mir-219-2	unconfirmed	CID 60843	hsa-let-7d	unconfirmed
CID 8223	hsa-mir-98	unconfirmed	CID 110635	hsa-mir-216b	unconfirmed
CID 19861	hsa-mir-659	unconfirmed	CID 2801	hsa-mir-744	unconfirmed
CID 71329	hsa-mir-100	unconfirmed	CID 216239	hsa-mir-1273e	unconfirmed
CID 47641	hsa-mir-150	unconfirmed	CID 71398	hsa-mir-526a-1	unconfirmed
CID 443980	hsa-mir-760	unconfirmed	CID 4201	hsa-mir-153-2	unconfirmed
CID 5574	hsa-mir-512-2	unconfirmed	CID 5281040	hsa-mir-548a-2	unconfirmed
CID 8969	hsa-mir-543	unconfirmed	CID 444020	hsa-mir-320a	unconfirmed
CID 5282415	hsa-mir-619	unconfirmed	CID 3025	hsa-mir-24-1	unconfirmed
CID 65833	hsa-mir-760	unconfirmed	CID 3019	hsa-mir-1226	unconfirmed
CID 1775	hsa-mir-520f	unconfirmed	CID 1125	hsa-mir-27a	unconfirmed
CID 3749	hsa-mir-1285-2	unconfirmed	CID 1349907	hsa-mir-642a	unconfirmed
CID 2905	hsa-mir-96	unconfirmed	CID 656719	hsa-mir-611	unconfirmed
CID 3180	hsa-mir-148a	unconfirmed	CID 2795	hsa-mir-711	unconfirmed
CID 5566	hsa-mir-646	unconfirmed	CID 23994	hsa-mir-614	unconfirmed
CID 4212	hsa-mir-18a	31063487	CID 4099	hsa-mir-708	unconfirmed
CID 82146	hsa-mir-490	unconfirmed	CID 5281106	hsa-mir-1302-6	unconfirmed

#### 3.2.1 5-Fu

5-Fu, one of the earliest anticancer drugs, can be fully absorbed by tumor cells. Moreover, 5-Fu can decrease tumor cell proliferation by interfering with the formation of DeoxyriboNucleic Acid (DNA) and RiboNucleic Acid (RNA) in tumor cells. It has been demonstrated that 5-Fu has considerable inhibitory effects on various cancer cells. Therefore, 5-Fu is frequently used as a positive control in anticancer drug effect experiments and clinical adjuvant treatment of gastric cancer (Longley et al., 2003). The first type of case studies’ results show that 9, 16 and 39 out of the top 10, 20 and 50 potential 5-Fu-associated miRNAs are confirmed in other literature or databases (See Table 3). The second type of case studies’ results show that 8, 15 and 39 out of the top 10, 20 and 50 potential 5-Fu-associated miRNAs are confirmed in other literature or databases (See Table 4). For example, 5-Fu is the most common chemotherapeutic agent for colorectal cancer. On the one hand, over-expression of hsa-miR-23a causes the resistance to 5-Fu in microsatellite instability colorectal cancer, which results in a diminished effect of 5-Fu chemotherapy (Shang et al., 2014). On the other hand, Ectopic expression of hsa-miR-23a increased the viability and survival of microsatellite stability colorectal cancer cells, thereby leading to the apoptosis of colorectal cancer cells (Li et al., 2015).

**TABLE 3:** Validation of the top 50 miRNAs associated with 5-Fu in the first type of case studies. The first column records the top 1–25 related miRNAs. The second column records the top 26–50 related miRNAs.

SM	miRNA	Evidence	SM	miRNA	Evidence
CID 3385	hsa-miR-151a	23220571	CID 3385	hsa-miR-126	26062749
CID 3385	hsa-miR-195	21947305	CID 3385	hsa-miR-128-1	23220571
CID 3385	hsa-let-7d	23220571	CID 3385	hsa-miR-337	unconfirmed
CID 3385	hsa-miR-195	21947305	CID 3385	hsa-miR-181c	unconfirmed
CID 3385	hsa-miR-125a	23220571	CID 3385	hsa-miR-30c-1	unconfirmed
CID 3385	hsa-miR-345	unconfirmed	CID 3385	hsa-miR-27a	23220571
CID 3385	hsa-miR-16-1	26198104	CID 3385	hsa-let-7a-1	23220571
CID 3385	hsa-miR-24-1	26198104	CID 3385	hsa-miR-139	27173050
CID 3385	hsa-miR-23b	23220571	CID 3385	hsa-miR-302b	26457704
CID 3385	hsa-miR-1226	26198104	CID 3385	hsa-let-7b	25789066
CID 3385	hsa-miR-151a	23220571	CID 3385	hsa-miR-26b	23220571
CID 3385	hsa-miR-132	23220571	CID 3385	hsa-miR-221	27501171
CID 3385	hsa-125b-1	unconfirmed	CID 3385	hsa-miR-338	28928082
CID 3385	hsa-let-7e	23220571	CID 3385	hsa-miR-130a	unconfirmed
CID 3385	hsa-miR-19a	23220571	CID 3385	hsa-miR-10b	22322955
CID 3385	hsa-miR-181a-1	unconfirmed	CID 3385	hsa-miR-204	27095441
CID 3385	hsa-miR-181b-1	unconfirmed	CID 3385	hsa-miR-26a-1	unconfirmed
CID 3385	hsa-miR-25	23220571	CID 3385	hsa-miR-92a-1	23220571
CID 3385	hsa-miR-106a	23220571	CID 3385	hsa-miR-299	31786874
CID 3385	hsa-miR-200c	23220571	CID 3385	hsa-miR-107	26636340
CID 3385	hsa-miR-22	25449431	CID 3385	hsa-miR-181a-2	24462870
CID 3385	hsa-miR-20a	23220571	CID 3385	hsa-miR-205	24396484
CID 3385	hsa-let-7d	23220571	CID 3385	hsa-miR-23a	23220571
CID 3385	hsa-miR-34b	unconfirmed	CID 3385	hsa-miR-199b	unconfirmed
CID 3385	hsa-miR-205	24396484	CID 3385	hsa-miR-93	23220571

**TABLE 4:** Validation of the top 50 miRNAs associated with 5-Fu in the second type of case studies. The first column records the top 1–25 related miRNAs. The second column records the top 26–50 related miRNAs.

SM	miRNA	Evidence	SM	miRNA	Evidence
CID 3385	hsa-miR-151a	23220571	CID 3385	hsa-miR-195	21947305
CID 3385	hsa-let-7d	23220571	CID 3385	hsa-miR-27a	23220571
CID 3385	hsa-miR-205	24396484	CID 3385	hsa-miR-204	27095441
CID 3385	hsa-miR-181a-2	24462870	CID 3385	hsa-miR-181a-1	unconfirmed
CID 3385	hsa-miR-23a	23220571	CID 3385	hsa-miR-25	23220571
CID 3385	hsa-miR-1226	26198104	CID 3385	hsa-miR-199b	unconfirmed
CID 3385	hsa-miR-181c	unconfirmed	CID 3385	hsa-miR-139	27173050
CID 3385	hsa-miR-151a	23220571	CID 3385	hsa-miR-195	21947305
CID 3385	hsa-miR-26a-1	unconfirmed	CID 3385	hsa-miR-132	23220571
CID 3385	hsa-miR-26b	23220571	CID 3385	hsa-miR-20a	23220571
CID 3385	hsa-miR-130a	unconfirmed	CID 3385	hsa-miR-126	26062749
CID 3385	hsa-miR-345	unconfirmed	CID 3385	hsa-125b-1	unconfirmed
CID 3385	hsa-miR-128-1	23220571	CID 3385	hsa-miR-200c	23220571
CID 3385	hsa-let-7d	23220571	CID 3385	hsa-miR-299	31786874
CID 3385	hsa-miR-181b-1	unconfirmed	CID 3385	hsa-miR-30c-1	unconfirmed
CID 3385	hsa-miR-205	24396484	CID 3385	hsa-miR-24-1	26198104
CID 3385	hsa-miR-125a	23220571	CID 3385	hsa-miR-93	23220571
CID 3385	hsa-miR-22	25449431	CID 3385	hsa-let-7e	23220571
CID 3385	hsa-miR-16-1	26198104	CID 3385	hsa-let-7b	25789066
CID 3385	hsa-miR-106a	23220571	CID 3385	hsa-miR-221	27501171
CID 3385	hsa-miR-23b	23220571	CID 3385	hsa-miR-19a	23220571
CID 3385	hsa-miR-338	28928082	CID 3385	hsa-miR-92a-1	23220571
CID 3385	hsa-miR-10b	22322955	CID 3385	hsa-miR-302b	26457704
CID 3385	hsa-let-7a-1	23220571	CID 3385	hsa-miR-107	26636340
CID 3385	hsa-miR-337	unconfirmed	CID 3385	hsa-miR-34b	unconfirmed

#### 3.2.2 5-Aza-CdR

5- Aza-CdR can bind to DNA methyltransferases to reduce methylation levels, reducing the biological activity of methyltransferase inhibitors and regulating gene expression. In clinical usage, 5-Aza-CdR is frequently used in clinical settings to treat diseases caused by gene variants (Do Amaral et al., 2019). Additionally, 5-Aza-CdR can suppress tumor cell proliferation via demethylation, making it one of the most potent inhibitors currently available *in vitro* (Lemaire et al., 2008). Meanwhile, 5-Aza-CdR can enhance the sensitivity of targeted drugs in non-small cell lung cancer chemotherapy, inhibit cell proliferation, accelerate the apoptosis of cancer cells, induce cell differentiation and activate quiescent anticancer cells in the human body. The first type of case studies’ results show that 8, 13 and 26 out of the top 10, 20 and 50 potential 5-Aza-CdR-associated miRNAs are confirmed in other literature or databases (See Table 5). The second type of case studies’ results show that 8, 14 and 28 out of the top 10, 20 and 50 potential 5-Aza-CdR-associated miRNAs are confirmed in other literature or databases (See Table 6). For example, quantitative methylation-specific Polymerase Chain Reaction analysis showed hypermethylation of the choline phosphoglyceride island adjacent to hsa-let-7e, and demethylation treatment with 5-Aza-CdR or transfection of pYr-let-7e-shRNA plasmid containing unmethylated hsa-let-7e DNA sequence could restore hsa-let-7e expression and partly reduce the chemoresistance (Cai et al., 2013).

**TABLE 5:** Validation of the top 50 miRNAs associated with 5-Aza-CdR in the first type of case studies. The first column records the top 1–25 related miRNAs. The second column records the top 26–50 related miRNAs.

SM	miRNA	Evidence	SM	miRNA	Evidence
CID 451668	hsa-miR-20a	23220571	CID 451668	hsa-miR-30a	unconfirmed
CID 451668	hsa-miR-320a	26198104	CID 451668	hsa-miR-107	23220571
CID 451668	hsa-miR-125a	23220571	CID 451668	hsa-miR-199b	24659709
CID 451668	hsa-miR-182	23220571	CID 451668	hsa-let-7a-1	unconfirmed
CID 451668	hsa-miR-204	unconfirmed	CID 451668	hsa-miR-92a-1	unconfirmed
CID 451668	hsa-miR-200b	23626803	CID 451668	hsa-miR-181a-1	23220571
CID 451668	hsa-miR-23a	unconfirmed	CID 451668	hsa-let-7e	22053057
CID 451668	hsa-let-7f-1	23220571	CID 451668	hsa-miR-26a-1	unconfirmed
CID 451668	hsa-let-7b	26708866	CID 451668	hsa-miR-1233-1	unconfirmed
CID 451668	hsa-miR-200c	23626803	CID 451668	hsa-miR-130a	23220571
CID 451668	hsa-miR-25	23220571	CID 451668	hsa-miR-30c-1	unconfirmed
CID 451668	hsa-miR-128-1	27705931	CID 451668	hsa-miR-22	23220571
CID 451668	hsa-miR-145	26198104	CID 451668	hsa-miR-301a	unconfirmed
CID 451668	hsa-miR-221	unconfirmed	CID 451668	hsa-let-7g	23220571
CID 451668	hsa-miR-19b-1	unconfirmed	CID 451668	hsa-miR-195	23333942
CID 451668	hsa-miR-197	unconfirmed	CID 451668	hsa-miR-302b	unconfirmed
CID 451668	hsa-let-7i	23220571	CID 451668	hsa-miR-26b	unconfirmed
CID 451668	hsa-miR-181b-1	unconfirmed	CID 451668	hsa-miR-205	unconfirmed
CID 451668	hsa-miR-338	unconfirmed	CID 451668	hsa-miR-218-1	unconfirmed
CID 451668	hsa-let-7d	26802971	CID 451668	hsa-miR-93	23220571
CID 451668	hsa-miR-139	unconfirmed	CID 451668	hsa-miR-124-1	unconfirmed
CID 451668	hsa-miR-328	unconfirmed	CID 451668	hsa-miR-15b	unconfirmed
CID 451668	hsa-miR-126	23220571	CID 451668	hsa-miR-10b	unconfirmed
CID 451668	hsa-miR-17	23220571	CID 451668	hsa-miR-128-2	unconfirmed
CID 451668	hsa-miR-19a	23220571	CID 451668	hsa-miR-27a	23220571

**TABLE 6:** Validation of the top 50 miRNAs associated with 5-Aza-CdR in the second type of case studies. The first column records the top 1–25 related miRNAs. The second column records the top 26–50 related miRNAs.

SM	miRNA	Evidence	SM	miRNA	Evidence
CID 451668	hsa-miR-20a	23220571	CID 451668	hsa-miR-92a-1	unconfirmed
CID 451668	hsa-miR-181b-1	unconfirmed	CID 451668	hsa-miR-125a	23220571
CID 451668	hsa-miR-205	unconfirmed	CID 451668	hsa-let-7b	26708866
CID 451668	hsa-miR-19a	23220571	CID 451668	hsa-miR-302b	unconfirmed
CID 451668	hsa-miR-181a-1	23220571	CID 451668	hsa-miR-30a	unconfirmed
CID 451668	hsa-miR-130a	23220571	CID 451668	hsa-miR-23b	23220571
CID 451668	hsa-let-7g	23220571	CID 451668	hsa-miR-199b	24659709
CID 451668	hsa-miR-200b	23626803	CID 451668	hsa-miR-128-2	unconfirmed
CID 451668	hsa-miR-126	23220571	CID 451668	hsa-miR-15b	unconfirmed
CID 451668	hsa-miR-320a	26198104	CID 451668	hsa-miR-124-1	unconfirmed
CID 451668	hsa-miR-30c-1	unconfirmed	CID 451668	hsa-miR-26b	unconfirmed
CID 451668	hsa-miR-328	unconfirmed	CID 451668	hsa-miR-128-1	27705931
CID 451668	hsa-let-7e	22053057	CID 451668	hsa-let-7a-1	unconfirmed
CID 451668	hsa-miR-10b	unconfirmed	CID 451668	hsa-miR-218-1	unconfirmed
CID 451668	hsa-let-7f-1	23220571	CID 451668	hsa-miR-200c	23626803
CID 451668	hsa-miR-221	unconfirmed	CID 451668	hsa-miR-26a-1	unconfirmed
CID 451668	hsa-miR-182	23220571	CID 451668	hsa-miR-338	unconfirmed
CID 451668	hsa-let-7i	23220571	CID 451668	hsa-miR-93	23220571
CID 451668	hsa-miR-195	23333942	CID 451668	hsa-miR-139	unconfirmed
CID 451668	hsa-miR-27a	23220571	CID 451668	hsa-miR-145	26198104
CID 451668	hsa-miR-204	unconfirmed	CID 451668	hsa-miR-107	23220571
CID 451668	hsa-miR-25	23220571	CID 451668	hsa-let-7d	26802971
CID 451668	hsa-miR-23a	unconfirmed	CID 451668	hsa-miR-19b-1	unconfirmed
CID 451668	hsa-let-7f-1	23220571	CID 451668	hsa-miR-22	23220571
CID 451668	hsa-miR-17	23220571	CID 451668	hsa-miR-197	unconfirmed

#### 3.2.3 E2

In addition to stimulating the growth and maintenance of the reproductive system, E2 exerts protective effects on cardiovascular and other organs. Specifically, E2 can reduce blood cholesterol levels by decreasing Low-Density Lipoprotein (LDL), increasing High-Density Lipoprotein (HDL) and boosting apolipoprotein content (Oh et al., 2019). Moreover, researchers are paying more attention to the anti-inflammatory, antioxidant and anti-apoptotic properties of E2 on cardiovascular diseases such as coronary heart disease and atherosclerosis, are getting more attention from researchers (Tse et al., 1999; Rachoń et al., 2002). The first type of case studies’ results show that 6, 14 and 29 out of the top 10, 20 and 50 potential E2-associated miRNAs are confirmed in other literature or databases (See Table 7). The second type of case studies’ results show that 4, 11 and 29 out of the top 10, 20 and 50 potential E2-associated miRNAs are confirmed in other literature or databases (See Table 8). For example, hsa-miR-23a could be negatively regulated by E2 in both myocardium and cultured cardiomyocytes. Moreover, hsa-miR-23a could directly down-regulate peroxisome proliferator-activated receptor γ coactivator-alpha (PGC-1α) expression in cardiomyocytes via binding to its 3′-untranslated regions, which implied that hsa-miR-23a could be critical for the down-regulation of PGC-1α under E2 deficiency (Sun et al., 2014).

**TABLE 7:** Validation of the top 50 miRNAs associated with E2 in the first type of case studies. The first column records the top 1–25 related miRNAs. The second column records the top 26–50 related miRNAs.

SM	miRNA	Evidence	SM	miRNA	Evidence
CID 5757	hsa-miR-183	unconfirmed	CID 5757	hsa-miR-181b-1	unconfirmed
CID 5757	hsa-let-7g	23220571	CID 5757	hsa-miR-19b-1	unconfirmed
CID 5757	hsa-miR-181a-2	unconfirmed	CID 5757	hsa-miR-141	unconfirmed
CID 5757	hsa-miR-125a	21914226	CID 5757	hsa-miR-15a	unconfirmed
CID 5757	hsa-miR-107	23220571	CID 5757	hsa-miR-17	23220571
CID 5757	hsa-miR-26b	24735615	CID 5757	hsa-miR-10b	23220571
CID 5757	hsa-miR-19a	29416771	CID 5757	hsa-miR-30a	29331043
CID 5757	hsa-miR-195	unconfirmed	CID 5757	hsa-let-7f-1	23220571
CID 5757	hsa-miR-128-2	23220571	CID 5757	hsa-miR-302b	23220571
CID 5757	hsa-miR-181a-1	unconfirmed	CID 5757	hsa-miR-199b	unconfirmed
CID 5757	hsa-miR-128-1	23220571	CID 5757	hsa-miR-181c	unconfirmed
CID 5757	hsa-miR-130a	unconfirmed	CID 5757	hsa-miR-106b	28422740
CID 5757	hsa-miR-338	22996663	CID 5757	hsa-miR-23a	23220571
CID 5757	hsa-let-7e	23220571	CID 5757	hsa-miR-9-2	23220571
CID 5757	hsa-miR-20a	21914226	CID 5757	hsa-miR-182	28678802
CID 5757	hsa-miR-200c	23220571	CID 5757	hsa-miR-139	unconfirmed
CID 5757	hsa-miR-27a	23220571	CID 5757	hsa-let-7b	23220571
CID 5757	hsa-miR-200b	23220571	CID 5757	hsa-miR-25	unconfirmed
CID 5757	hsa-miR-221	21057537	CID 5757	hsa-miR-218-1	unconfirmed
CID 5757	hsa-miR-151a	unconfirmed	CID 5757	hsa-miR-22	24715036
CID 5757	hsa-miR-204	29789714	CID 5757	hsa-miR-15b	23220571
CID 5757	hsa-miR-106a	unconfirmed	CID 5757	hsa-miR-130a	unconfirmed
CID 5757	hsa-miR-205	unconfirmed	CID 5757	hsa-miR-23b	23220571
CID 5757	hsa-miR-92a-1	unconfirmed	CID 5757	hsa-miR-26a-1	unconfirmed
CID 5757	hsa-miR-130b	unconfirmed	CID 5757	hsa-miR-30c-1	23220571

**TABLE 8:** Validation of the top 50 miRNAs associated with E2 in the second type of case studies. The first column records the top 1–25 related miRNAs. The second column records the top 26–50 related miRNAs.

SM	miRNA	Evidence	SM	miRNA	Evidence
CID 5757	hsa-miR-183	unconfirmed	CID 5757	hsa-miR-19a	29416771
CID 5757	hsa-miR-30c-1	23220571	CID 5757	hsa-miR-19b-1	unconfirmed
CID 5757	hsa-miR-15a	unconfirmed	CID 5757	hsa-miR-125a	21914226
CID 5757	hsa-miR-181a-1	unconfirmed	CID 5757	hsa-miR-15b	23220571
CID 5757	hsa-let-7f-1	23220571	CID 5757	hsa-miR-128-2	23220571
CID 5757	hsa-miR-181b-1	unconfirmed	CID 5757	hsa-miR-20a	21914226
CID 5757	hsa-miR-205	unconfirmed	CID 5757	hsa-miR-26b	24735615
CID 5757	hsa-miR-181a-2	unconfirmed	CID 5757	hsa-miR-10b	23220571
CID 5757	hsa-miR-9-2	23220571	CID 5757	hsa-miR-181c	unconfirmed
CID 5757	hsa-miR-23a	23220571	CID 5757	hsa-miR-22	24715036
CID 5757	hsa-miR-128-1	23220571	CID 5757	hsa-miR-139	unconfirmed
CID 5757	hsa-let-7e	23220571	CID 5757	hsa-miR-106a	unconfirmed
CID 5757	hsa-let-7b	23220571	CID 5757	hsa-miR-141	unconfirmed
CID 5757	hsa-miR-130a	unconfirmed	CID 5757	hsa-let-7g	23220571
CID 5757	hsa-miR-338	22996663	CID 5757	hsa-miR-107	23220571
CID 5757	hsa-miR-30a	29331043	CID 5757	hsa-miR-23b	23220571
CID 5757	hsa-miR-302b	23220571	CID 5757	hsa-miR-195	unconfirmed
CID 5757	hsa-miR-130b	unconfirmed	CID 5757	hsa-miR-27a	23220571
CID 5757	hsa-miR-106b	28422740	CID 5757	hsa-miR-25	unconfirmed
CID 5757	hsa-miR-199b	unconfirmed	CID 5757	hsa-miR-204	29789714
CID 5757	hsa-miR-200b	23220571	CID 5757	hsa-miR-221	21057537
CID 5757	hsa-miR-182	28678802	CID 5757	hsa-miR-151a	unconfirmed
CID 5757	hsa-miR-26a-1	unconfirmed	CID 5757	hsa-miR-218-1	unconfirmed
CID 5757	hsa-miR-17	23220571	CID 5757	hsa-miR-130a	unconfirmed
CID 5757	hsa-miR-200c	23220571	CID 5757	hsa-miR-92a-1	unconfirmed

## 4 Discussion

Deep learning offers a wide range of applications in major areas of computer science, such as computer vision, natural language processing and machine translation. More effective models can be obtained by adding hidden layers to standard neural networks. Deep learning also contributes to medication development and precision medicine by predicting potential SM-miRNA associations. Furthermore, deep learning models have more hidden layer nodes than conventional neural networks. The number of hidden layers can even reach ten for extremely complex problems. After multiple layers of calculation, the results of deep learning-based algorithms are often closer to the actual situation than those of traditional machine learning-based algorithms. Initially, we utilized matrix decomposition to reduce noise from known SM-miRNA associations. Then, the layer attention mechanism was introduced to the deep learning model, which significantly improved the performance of our model by integrating the SM-miRNA association feature vectors used for calculation.

GCNLASMMA is a model of a neural network with numerous hidden layers. Multiple layers computations allowed the results to completely consider known features and avoid overfitting. The attention mechanism extracted vital information from each layer of the neural network. Besides, the matrix decomposition module reduced the noise of known SM-miRNA associations, significantly enhancing GCN’s performance. GCNLASMMA was an attempt to identify potential SM-miRNA associations using deep learning. The advantages above enabled GCNLASMMA to accurately anticipate potential SM-miRNA associations.

Deep learning’s spectacular performance is contingent on a vast number of known SM-miRNA associations. The number of known SM-miRNA associations utilized in this investigation was apparently insufficient to fulfill GCNLASMMA. Therefore, the performance of GCNLASMMA was still unsatisfactory. In addition, the parameters used in GCNLASMMA may not be ideal. Moreover, the construction of heterogeneous networks will yield better results if other biological information, such as long non-coding RNA or disease, is utilized. These factors will motivate researchers to develop more effective deep learning models to predict potential SM-miRNA associations using more trustworthy biological datasets.

## Data Availability

The Python code and datasets of GCNLASMMA are publicly available at https://github.com/1054366388/GCNLASMMA.
